# Supplementary material for: Application of open domain adaptive models in image annotation and classification
Source: PLoS One. 2025 May 14;20(5):e0322836. doi: 10.1371/journal.pone.0322836 (PMC12077773; doi:10.1371/journal.pone.0322836)
Supplement: S1 File — (DOCX) [file pone.0322836.s001.docx]

**Figure 8 Test results for optimal thresholds $\beta$ and $\psi$**

| Threshold (β or ψ) | ImageNet-ACC-K (%) | COCO-ACC-K (%) | ImageNet-ACC-UNK (%) | COCO-ACC-UNK (%) |
| --- | --- | --- | --- | --- |
| β=0.10 | 40.25 | 40.34 | 90.47 | 80.16 |
| β=0.20 | 50.67 | 50.42 | 85.79 | 75.33 |
| β=0.30 | 60.32 | 60.21 | 80.63 | 70.14 |
| β=0.40 | 65.58 | 65.74 | 75.29 | 65.18 |
| β=0.60 | 67.45 | 67.43 | 70.27 | 60.19 |
| Ψ=0.10 | 10.23 | 10.31 | 60.18 | 50.12 |
| Ψ=0.30 | 40.22 | 40.47 | 85.29 | 75.14 |
| Ψ=0.50 | 70.13 | 70.11 | 95.21 | 90.16 |
| Ψ=0.70 | 89.37 | 89.22 | 96.11 | 94.12 |

**Figure 9 Test results of dynamic weight $W$**

| Iterations | W=1.0(%) | W=0.8(%) | W=0.6(%) | W=0.4(%) | W=0.2(%) |
| --- | --- | --- | --- | --- | --- |
| 50 | 40.12 | 38.45 | 35.78 | 30.54 | 25.89 |
| 100 | 60.27 | 58.39 | 55.62 | 48.71 | 40.23 |
| 150 | 70.48 | 68.57 | 65.34 | 55.12 | 48.67 |
| 200 | 75.61 | 73.49 | 70.18 | 60.35 | 50.74 |
| 250 | 83.78 | 81.54 | 78.62 | 65.18 | 55.32 |
| 300 | 85.23 | 83.76 | 80.94 | 68.57 | 58.43 |
| 350 | 87.54 | 85.67 | 82.34 | 70.18 | 60.17 |
| 400 | 88.91 | 86.74 | 83.29 | 72.45 | 62.43 |
| 450 | 89.78 | 87.54 | 84.12 | 73.49 | 63.18 |
| 500 | 89.89 | 87.65 | 84.43 | 74.12 | 63.87 |

**Figure 10 PR curves and area test results for each module**

| Recall | ODA Precision (%) | ODA-DTC Precision (%) | ODA-DTC-Dynamic Weight Precision (%) | ODA-DTC-Dynamic Weight-Subdomain Alignment Precision (%) |
| --- | --- | --- | --- | --- |
| 0 | 100 | 100.00 | 100.00 | 100.00 |
| 0.2 | 85.47 | 90.67 | 95.78 | 96.45 |
| 0.4 | 72.36 | 80.12 | 89.32 | 92.34 |
| 0.6 | 60.18 | 70.45 | 81.27 | 86.45 |
| 0.8 | 45.67 | 58.23 | 72.84 | 80.67 |
| 1.0 | 30.24 | 45.12 | 60.34 | 73.12 |

**Figure 13 Image confusion matrix results of two models**

| Actual class \ model | Car | Train | Horse | Apple | Book | Bee | House |
| --- | --- | --- | --- | --- | --- | --- | --- |
| Car (ADDA) | 99 | 63 | 23 | 21 | 27 | 42 | 28 |
| Car (Our Model) | 99 | 63 | 65 | 41 | 27 | 26 | 29 |
| Train (ADDA) | 67 | 95 | 21 | 27 | 31 | 47 | 19 |
| Train (Our Model) | 67 | 95 | 21 | 53 | 31 | 47 | 19 |
| Horse (ADDA) | 67 | 21 | 92 | 36 | 31 | 29 | 48 |
| Horse (Our Model) | 21 | 12 | 92 | 29 | 39 | 22 | 38 |
| Apple (ADDA) | 32 | 12 | 52 | 65 | 36 | 18 | 38 |
| Apple (Our Model) | 36 | 65 | 18 | 89 | 31 | 33 | 37 |
| Book (ADDA) | 72 | 36 | 29 | 29 | 81 | 18 | 37 |
| Book (Our Model) | 36 | 54 | 22 | 36 | 95 | 27 | 27 |
| Bee (ADDA) | 52 | 47 | 29 | 29 | 37 | 75 | 48 |
| Bee (Our Model) | 38 | 46 | 27 | 32 | 45 | 97 | 19 |
| House (ADDA) | 42 | 19 | 24 | 38 | 37 | 37 | 47 |
| House (Our Model) | 29 | 11 | 27 | 25 | 27 | 17 | 96 |
