# Supplementary material for: Application of open domain adaptive models in image annotation and classification
Source: PLoS One. 2025 May 14;20(5):e0322836. doi: 10.1371/journal.pone.0322836 (PMC12077773; doi:10.1371/journal.pone.0322836)
Supplement: Attachment — Submitted filename: Response to reviewers comments.docx [file pone.0322836.s003.docx]

Review Comments to the Author

Please use the space provided to explain your answers to the questions above. You may also include additional comments for the author, including concerns about dual publication, research ethics, or publication ethics. (Please upload your review as an attachment if it exceeds 20,000 characters)

Reviewer #1:

Abstract - Lack of Clear Motivation and Problem Definition, Unclear Novelty, Methodological Clarity, Ambiguity in Performance Metrics, Absence of Practical Implications and Language Precision and Flow.

Reply: Thank you very much for your kind comments. The abstract section has been rewritten, and rhe revised abstract mainly including the research background, motivation, problem, novelty, the proposed method, the key findings, conclusion and contributions and so on.

Justification needed for "Authors might choose conventional methods over recent learning-based methods for several reasons, particularly when developing models for open-domain adaptive image annotation and classification. "

Reply: Thank you for your review comments. A number of explanations have been provided to clearly illustrate the rationale for selecting methods when developing open domain adaptive image labeling and classification models. Please see details as follows:

The main reason for choosing the domain adaptive algorithm is that the significant difference in the data distribution between the source and target domains leads to the poor performance of traditional supervised learning methods in the target domain, especially in the open-domain scenarios, where the target domain contains new categories that have not been seen in the source domain, which makes the generalization ability of the model challenging. The domain adaptive algorithm solves the problem of inconsistent distribution of training and test data by aligning the feature distributions of the source and target domains, thus improving the classification accuracy of the model on the target domain ^[17-19]^.

In open-domain scenarios, target-domain data usually contain unknown categories whose distribution is significantly different from that of the source domain. Fixed threshold models are difficult to balance the classification performance of known and unknown categories, which may lead to misclassification of unknown category data DTC effectively improves the model's ability to recognize unknown categories by dynamically adjusting the threshold according to the sample importance.

Some recent study can be included.

Reply: Thank you for your review comments. The introduction section has been revised to add the latest literature on image classification related technologies in the open set field in 2023-2024, as follows:

Parmar J et al. In order to enhance the effectiveness of open machine learning techniques in open set adaptive domain image classification, the research Confucians assembled natural language processing techniques and proposed a novel image classification method. The experimental results show that the accuracy of open set adaptive image classification under this method is higher than the traditional method and the image classification is more adaptive ^[14]^.Chen H et al. proposed a new method for stable classification of hyperspectral images, which is based on superpixel principal component analysis and random patch network. Not only can the data-driven approach be utilized, but it can also be applied to efficiently take into account more global and local spectral knowledge at the hyperpixel level. Test results on several open-set domain image classification datasets show that the method significantly outperforms several current state-of-the-art methods ^[15]^.

[14]Parmar J, Chouhan S, Raychoudhury V, Rathore S. "Open-world machine learning: applications, challenges, and opportunities." ACM Comput. Surv., vol. 55, no. 10, pp. 1-37, February 2023, DOI: 10.1145/3561381.

[15]Chen H, Wang T, Chen T, Deng W. "Hyperspectral image classification based on fusing S3-PCA, 2D-SSA and random patch network." Remote Sens., vol. 15, no. 13, pp. 3402-3407, July 2023, DOI: 10.3390/rs15133402.

Highlights of works must be given.

Reply: Thank you for your review comments. The introductory section of the manuscript has been revised to clarify the brightness of the study, as modified below:

An optimized open-domain adaptive image annotation classification model (ODA-IAC) is proposed, whose main highlights include: the introduction of a domain adaptive algorithm (DA) to reduce the differences in feature distributions between the source and target domains; the combination of a dynamic threshold control module (DTC), which improves the model's classification accuracy of unknown categories by real-time adjusting of thresholds to adapt to the changes in the distribution of samples; and the adoption of a channel attention mechanism ( CAM) to enhance the expression ability of important features; and adding the subdomain alignment strategy (SAS) to further refine the feature distribution within the target domain and improve the model's adaptability to complex scenes

Novelty should be highlighted.

Reply: Thank you for your review comments. The introduction section has been revised to clarify the novelty of the study, as modified below:

The novelty of the research lies in the integrated use of dynamic threshold control, channel attention mechanism and subdomain alignment strategy, which solves the performance bottleneck of traditional domain adaptive algorithms in open-domain scenarios through the organic combination of modules.

Summary of limitations of the existing must be given.

Reply: Thank you for your review comments. The introductory section of the manuscript has been revised to clarify the limitations of the existing study, as modified below:

In summary, in recent years, image annotation and classification techniques play an important role in the field of computer vision, and are widely used in scenarios such as medical image analysis, intelligent surveillance and image retrieval. However, traditional supervised learning methods are highly dependent on the distribution of the training data, and when faced with the difference in data distribution between the source and target domains, especially in open-domain scenarios, the target domain data may contain new categories that have not been seen in the source domain, which results in insufficient generalization ability of the model and a significant decrease in classification accuracy. These limitations indicate that existing techniques are significantly inadequate for open-domain image annotation and classification tasks.

Evaluate the model’s performance on data migration across domains, specifically when it is applied to classify target-domain images based on source-domain training and vice versa.

Reply: Thank you for your review comments. In order to evaluate the performance of the model in cross-domain data migration, the study conducts migration tests between source and target domains respectively, i.e., training the model on the source domain data and classifying the target domain images as well as training the model on the target domain data and classifying the source domain images, and the test results are shown in Table 1, using the checking accuracy rate, the recall rate, the F1, the runtime, and the annotation consistency as test metrics.

**Table 1 Test results of indicators for different models**

| Categorized object | Model | P/% | R/% | F1/% | Running time/s | Annotation consistency | Efficiency/% | Reference |
| --- | --- | --- | --- | --- | --- | --- | --- | --- |
| Known areas | DANN | 78.6 | 81.2 | 79.9 | 4.2 | 0.86 | 89.72 | [29] |
|  | CADA | 83.5 | 85.7 | 84.6 | 5 | 0.81 | 87.34 | [30] |
|  | SEDA | 79.6 | 85.1 | 82.3 | 3.1 | 0.89 | 89.19 | [31] |
|  | ODA-IAC | 89.6 | 90.5 | 90 | 1.8 | 0.91 | 91.28 | This study |
| Unknown areas | DANN | 82.1 | 84.6 | 83.3 | 4.2 | 0.85 | 79.34 | [29] |
|  | CADA | 83.4 | 85.7 | 84.5 | 4.1 | 0.89 | 83.52 | [30] |
|  | SEDA | 79.6 | 87.9 | 83.7 | 2.5 | 0.91 | 84.41 | [31] |
|  | ODA-IAC | 89.3 | 90.7 | 90.2 | 1.2 | 0.92 | 89.56 | This study |

In Table 1, the research proposed model performs well in the performance test of cross-domain data migration, and its check-accuracy, recall, and F1 value are significantly higher than those of the other models, both in the classification tasks of known and unknown domains. Among them, in the known domain, the study of the proposed model achieves 89.6% and 90.5% of check accuracy and recall, respectively, with an F1 value of 90%, an annotation consistency of 0.91, and the shortest running time of only 1.8 seconds. As for the unknown domain, the checking accuracy and recall are 89.3% and 90.7%, respectively, with an F1 value of 90.2%, and the annotation consistency is further improved to 0.92 with the shortest running time of only 1.2 seconds. In contrast, the performance of other models such as DANN and CADA is competitive, but they have longer running time and lower annotation consistency, especially in the migration test of unknown domains, with the highest F1 value of only 84.5%. In addition, the SEDA model performs relatively well in annotation consistency, but its F1 value and runtime still do not reach the level of the models mentioned in the study.

Assess consistency in annotations across similar images in both known and unknown domains to ensure stable performance.

Reply: Thank you for your review comments. Table 1 in the experimental section of Section 2 of the manuscript has been modified to give annotated consistency results for similar test images, as modified below:

**Table 1 Test results of indicators for different models**

| Categorized object | Model | P/% | R/% | F1/% | Running time/s | Annotation consistency | Efficiency/% | Reference |
| --- | --- | --- | --- | --- | --- | --- | --- | --- |
| Known areas | DANN | 78.6 | 81.2 | 79.9 | 4.2 | 0.86 | 89.72 | [29] |
|  | CADA | 83.5 | 85.7 | 84.6 | 5 | 0.81 | 87.34 | [30] |
|  | SEDA | 79.6 | 85.1 | 82.3 | 3.1 | 0.89 | 89.19 | [31] |
|  | ODA-IAC | 89.6 | 90.5 | 90 | 1.8 | 0.91 | 91.28 | This study |
| Unknown areas | DANN | 82.1 | 84.6 | 83.3 | 4.2 | 0.85 | 79.34 | [29] |
|  | CADA | 83.4 | 85.7 | 84.5 | 4.1 | 0.89 | 83.52 | [30] |
|  | SEDA | 79.6 | 87.9 | 83.7 | 2.5 | 0.91 | 84.41 | [31] |
|  | ODA-IAC | 89.3 | 90.7 | 90.2 | 1.2 | 0.92 | 89.56 | This study |

Measure the time taken for the model to classify or annotate images, especially in unknown domains. Efficiency is particularly important if the model is intended for real-time applications.

Reply: Thank you for your review comments. The processing times of the model in the unknown image domain has been provided in Table 1.

In reviewer point of view "relying solely on classification accuracy may be insufficient because this metric alone does not provide a comprehensive view of the model's performance, especially in the context of open-domain and adaptive tasks. "

Reply: Thank you for your review comments. The manuscript does not use only classification accuracy, but also employs Precision, Recall, F1-Score, mean average precision (mAP), and runtime as additional metrics. Among them, Precision is used to measure the proportion of positive samples predicted by the model that are correctly predicted, Recall is used to measure the ability of the model to correctly identify actual positive samples, F1-Score balances the weights of Precision and Recall, reflecting the overall classification ability of the model, and AP, by evaluating the area under the Precision-Recall curve, is used.

The model might perform well on certain types of domain shifts or specific datasets but may struggle with more complex, real-world shifts that introduce significant variability (e.g., varying lighting, resolutions, or object occlusions).

Reply: Thank you for your review comments. Section 2.2 of the manuscript has been revised to add the results of model testing under different lighting conditions, as modified below:

**Table 2 Comparison of image transmission effects between domains under different lighting conditions**

| Lighting conditions | Object | Model | ACC-K/% | ACC-UNK/% | Reference |
| --- | --- | --- | --- | --- | --- |
| Low light | Source Domain - Target Domain | DANN | 76.3 | 80.5 | [29] |
|  |  | CADA | 81.2 | 86.7 | [30] |
|  |  | SEDA | 86.7 | 90.1 | [31] |
|  |  | ODA-IAC | 89.6 | 93.5 | This study |
|  | Target Domain - Source Domain | DANN | 74.1 | 81.2 | [29] |
|  |  | CADA | 79.6 | 85.7 | [30] |
|  |  | SEDA | 84.5 | 89.9 | [31] |
|  |  | ODA-IAC | 89.2 | 92.1 | This study |
| Normal light | Source Domain - Target Domain | DANN | 90.4 | 89.4 | [29] |
|  |  | CADA | 89.5 | 90.5 | [30] |
|  |  | SEDA | 91.6 | 91.3 | [31] |
|  |  | ODA-IAC | 93.2 | 92.8 | This study |
|  | Target Domain - Source Domain | DANN | 89.8 | 91.2 | [29] |
|  |  | CADA | 91.1 | 92.4 | [30] |
|  |  | SEDA | 92.7 | 90.7 | [31] |
|  |  | ODA-IAC | 94.5 | 93.3 | This study |
| Strong light | Source Domain - Target Domain | DANN | 84.3 | 87.4 | [29] |
|  |  | CADA | 89.7 | 85.2 | [30] |
|  |  | SEDA | 90.2 | 89.3 | [31] |
|  |  | ODA-IAC | 90.9 | 90.2 | This study |
|  | Target Domain - Source Domain | DANN | 89.4 | 88.2 | [29] |
|  |  | CADA | 88.3 | 89.3 | [30] |
|  |  | SEDA | 90.1 | 90.2 | [31] |
|  |  | ODA-IAC | 90.9 | 91.5 | This study |

If the model is primarily tested on a few datasets with relatively mild domain shifts, its effectiveness in handling more extreme, real-world scenarios could be limited.

Reply: Thank you for your review comments. Section 2.2 of the manuscript has been revised to supplement the more realistic and larger dataset for testing with the following modifications:

The study used a larger real dataset as the source of the test data, namely Domain Adaptation Dataset (DomainNet).DomainNet contains images from six different domains, namely clip art, sketches, paintings, real photographs, information icons and sketches. In total, there are about 600,000 images, covering 345 categories, which include animals, daily objects, transportation, etc. The DomainNet is also introduced with a more advanced domain adversarial approach.

The model’s performance may be strongly tied to the quality and relevance of the source domain. If the source domain is significantly different from the target domain, or if it lacks diverse classes, the model might struggle with adaptation, resulting in poor generalization.

Reply: Thank you for your review comments. Section 2.2 of the manuscript has been revised to add data consistency between source domains and target collars in the data sources, as modified below:
The VisDA dataset contains both the source domain (synthetic images) and the target domain (real images), covering 12 widely distributed categories to maximize the diversity and representativeness between the source and target domains^[32-34]^. In addition, the study reduces the impact of data bias and uneven distribution of categories on model performance by preprocessing and balanced sampling of the source domain data to ensure that the model trained in the source domain can adapt to the uncommon or low-frequency categories in the target domain, which improves the model's generalization ability and robustness.

Data biases or imbalances in the source domain could impact how well the model performs when encountering less common or underrepresented classes in the target domain.

Reply: Thank you for your review comments. It was verified that no similar phenomenon occurs in the manuscript, i.e., there are less common or underrepresented classes in the target domain, and therefore, no modifications were made for the time being.

While the study may include comparisons with conventional methods, it might lack a thorough comparison with recent learning-based domain adaptation or open-set classification methods. This can make it difficult to assess how the proposed model truly stands up against state-of-the-art techniques.

Reply: Thank you for your review comments. Section 2.2 of the manuscript has been revised to add the latest technology for comparison, as modified below:

The more advanced Domain-Adversarial Neural Network (DANN), Conditional Adversarial Domain Adaptation (CADA), and self-integrating for Visual Domain Adaptation (elf-) are also introduced. Ensembling for Visual Domain Adaptation (SEDA). ACC-K and ACC-UNK are used as indicators to test the migration effect of the four models between the source domain image and the target domain image under different lighting conditions, and the test results are shown in Table 2.

**Table 2 Comparison of image transmission effects between domains under different lighting conditions**

| Lighting conditions | Object | Model | ACC-K/% | ACC-UNK/% | Reference |
| --- | --- | --- | --- | --- | --- |
| Low light | Source Domain - Target Domain | DANN | 76.3 | 80.5 | [29] |
|  |  | CADA | 81.2 | 86.7 | [30] |
|  |  | SEDA | 86.7 | 90.1 | [31] |
|  |  | ODA-IAC | 89.6 | 93.5 | This study |
|  | Target Domain - Source Domain | DANN | 74.1 | 81.2 | [29] |
|  |  | CADA | 79.6 | 85.7 | [30] |
|  |  | SEDA | 84.5 | 89.9 | [31] |
|  |  | ODA-IAC | 89.2 | 92.1 | This study |
| Normal light | Source Domain - Target Domain | DANN | 90.4 | 89.4 | [29] |
|  |  | CADA | 89.5 | 90.5 | [30] |
|  |  | SEDA | 91.6 | 91.3 | [31] |
|  |  | ODA-IAC | 93.2 | 92.8 | This study |
|  | Target Domain - Source Domain | DANN | 89.8 | 91.2 | [29] |
|  |  | CADA | 91.1 | 92.4 | [30] |
|  |  | SEDA | 92.7 | 90.7 | [31] |
|  |  | ODA-IAC | 94.5 | 93.3 | This study |
| Strong light | Source Domain - Target Domain | DANN | 84.3 | 87.4 | [29] |
|  |  | CADA | 89.7 | 85.2 | [30] |
|  |  | SEDA | 90.2 | 89.3 | [31] |
|  |  | ODA-IAC | 90.9 | 90.2 | This study |
|  | Target Domain - Source Domain | DANN | 89.4 | 88.2 | [29] |
|  |  | CADA | 88.3 | 89.3 | [30] |
|  |  | SEDA | 90.1 | 90.2 | [31] |
|  |  | ODA-IAC | 90.9 | 91.5 | This study |

In Table 2, under low light conditions, the ACC-K and ACC-UNK values of the proposed model of the study reached 89.6% and 93.5%, respectively, which were significantly higher than those of DANN (76.3% and 80.5%), CADA (81.2% and 86.7%), and SEDA (86.7% and 90.1%), reflecting the robustness and adaptability in low light conditions. Under normal light conditions, the ACC-K and ACC-UNK values of the proposed model are further improved to 93.2% and 92.8%, respectively, which are further optimized compared with the highest values of the other models (91.6% and 91.3% for SEDA), demonstrating strong adaptability in typical light environments. Under strong light conditions, the ACC-K and ACC-UNK values of the proposed model reached 90.9% and 91.5%, respectively, which still dominated the performance compared with the other compared models, especially the ACC-UNK value of CADA decreased to 85.2% under strong light, while the proposed model maintained a high stability under strong light conditions. Taken together, the proposed model can better adapt to changes in lighting conditions during cross-domain migration and shows stronger generalization ability and robustness, thanks to the effective combination of dynamic threshold adjustment and subdomain alignment strategy in the model, which results in finer feature extraction and better classification of images in different lighting environments. In addition, in order to adapt to real-time scenarios, the study integrates a parallel computing strategy, which ensures the stability and low latency of the model when processing high throughput data through GPU acceleration and distributed inference techniques.

The study may not address practical deployment issues, such as how well the model scales to large, real-world datasets or handles real-time annotation and classification needs.

Reply: Thank you for your review comments. Section 2.2 of the manuscript has been revised, supplemented with tests using a real migration dataset and a more in-depth analysis of the results, as modified below:

The study used a larger real dataset as the source of the test data, namely Domain Adaptation Dataset (DomainNet).DomainNet contains images from six different domains, namely clip art, sketches, paintings, real photographs, information icons and sketches. In total, there are about 600,000 images, covering 345 categories, which include animals, daily objects, transportation, etc. The DomainNet is also introduced with a more advanced domain adversarial approach.

Taken together, the proposed model can better adapt to changes in lighting conditions during cross-domain migration and shows stronger generalization ability and robustness, thanks to the effective combination of dynamic threshold adjustment and subdomain alignment strategy in the model, which results in finer feature extraction and better classification of images in different lighting environments. In addition, in order to adapt to real-time scenarios, the study integrates a parallel computing strategy, which ensures the stability and low latency of the model when processing high throughput data through GPU acceleration and distributed inference techniques.

Open-set recognition remains challenging, as the model must not only classify known images correctly but also accurately detect and separate unknown classes. False positives in unknown class detection can lead to reduced model reliability, while false negatives can compromise the model’s ability to recognize new patterns.

Reply: Thank you for your review comments. Table 1 in Section 2.1 of the manuscript has been modified to additionally test the model's effectiveness in classifying images of unknown domains, with the following modifications:

In order to evaluate the performance of the model in cross-domain data migration, the study conducts migration tests between source and target domains respectively, i.e., training the model on the source domain data and classifying the target domain images as well as training the model on the target domain data and classifying the source domain images, and the test results are shown in Table 1, using the checking accuracy rate, the recall rate, the F1, the runtime, and the annotation consistency as test metrics.

**Table 1 Test results of indicators for different models**

| Categorized object | Model | P/% | R/% | F1/% | Running time/s | Annotation consistency | Efficiency/% | Reference |
| --- | --- | --- | --- | --- | --- | --- | --- | --- |
| Known areas | DANN | 78.6 | 81.2 | 79.9 | 4.2 | 0.86 | 89.72 | [29] |
|  | CADA | 83.5 | 85.7 | 84.6 | 5 | 0.81 | 87.34 | [30] |
|  | SEDA | 79.6 | 85.1 | 82.3 | 3.1 | 0.89 | 89.19 | [31] |
|  | ODA-IAC | 89.6 | 90.5 | 90 | 1.8 | 0.91 | 91.28 | This study |
| Unknown areas | DANN | 82.1 | 84.6 | 83.3 | 4.2 | 0.85 | 79.34 | [29] |
|  | CADA | 83.4 | 85.7 | 84.5 | 4.1 | 0.89 | 83.52 | [30] |
|  | SEDA | 79.6 | 87.9 | 83.7 | 2.5 | 0.91 | 84.41 | [31] |
|  | ODA-IAC | 89.3 | 90.7 | 90.2 | 1.2 | 0.92 | 89.56 | This study |

In Table 1, the research proposed model performs well in the performance test of cross-domain data migration, and its check-accuracy, recall, and F1 value are significantly higher than those of the other models, both in the classification tasks of known and unknown domains. Among them, in the known domain, the study of the proposed model achieves 89.6% and 90.5% of check accuracy and recall, respectively, with an F1 value of 90%, an annotation consistency of 0.91, and the shortest running time of only 1.8 seconds. As for the unknown domain, the checking accuracy and recall are 89.3% and 90.7%, respectively, with an F1 value of 90.2%, and the annotation consistency is further improved to 0.92 with the shortest running time of only 1.2 seconds. In contrast, the performance of other models such as DANN and CADA is competitive, but they have longer running time and lower annotation consistency, especially in the migration test of unknown domains, with the highest F1 value of only 84.5%. In addition, the SEDA model performs relatively well in annotation consistency, but its F1 value and runtime still do not reach the level of the models mentioned in the study.

Reviewer #2:

The manuscript has been drafted well.

The authors can consider the following comments

1. The specification of the Images used should be mentioned.

Reply: Thank you for your review comments. The image specifications used in this study include RGB three-channel images with a resolution of 224 × 224 pixels in JPEG format, derived from ImageNet and COCO datasets. These images were normalized, including resizing and pixel normalization, to ensure adaptation to model input requirements.

2. Feature extraction details have to be given elaborately.

Reply: Thank you very much for your review comments. Feature extraction details have been given as follows:

In terms of process, firstly, a convolutional neural network is utilized to extract multi-scale image features from low to high levels to provide rich basic information for the subsequent modules. Secondly, the channel attention mechanism is introduced to enhance the expression of important features through adaptive pooling and weighting operations to avoid the interference of redundant information on feature extraction. In addition, the dynamic threshold control module adjusts the feature extraction weights in real time according to the importance of the image samples, so as to optimize the expression ability of the features in diverse scenes.

3. Details about Training and testing the proposed model is missing.

Reply: Thank you very much for your review comments. The details about Training and testing the proposed model have been provided as follows:

In order to validate the model performance, the study uses ImageNet and COCO as the open-set dataset, and divides the data into training and test sets, where the training set accounts for 80% and the test set accounts for 20%. The training process was carried out in an Intel Core i7 CPU and NVIDIA GeForce GPU environment with 32 GB of RAM, developed in Python, with a learning rate set to 0.002, a batch size of 64, and 200 training rounds. The model was trained using cross-entropy loss function and Adam optimizer through dynamic threshold adjustment and subdomain alignment optimization. In the testing phase, the model is evaluated with classification accuracy, checking accuracy, recall and F1 value, while comparing the performance of traditional domain adaptive models to ensure the comprehensiveness and reliability of the results.

4. Is the accuracy mentioned in table 2 an average accuracy?

Reply: Thank you for your review comments. The accuracies in Table 2 are not average accuracies, ACC-K is the classification accuracy of known images, and ACC-UNK is the classification accuracy of unknown images.

5. The explanation about the proposed model can be even more descriptive

Reply: Thank you for your review comments. More explanation of the proposed modes is shown as follows:

In Figure 2, DTC-ODA can be divided into four major modules, i.e., domain module, channel attention module, labeling module and adaptive module. The domain module is used to input source and target domain data for feature generation and distribution alignment; the channel attention module improves the expression of key features through weight addition; the annotation module realizes the accurate annotation of known and unknown categories through the dynamic thresholding mechanism; and the adaptive module dynamically adjusts the inter-domain differences in combination with the sub-domain alignment strategy to enhance the feature alignment effect of the target domain data.

6. Clarify the scope of the research, including the specific aspects.

Reply: Thank you for your review comments. The scope of this study focuses on open-domain image annotation and classification tasks, specifically including the effective alignment of source and target domain features, the efficient utilization of unlabeled data in the target domain, the impact of dynamic threshold adjustment on classification performance, and the refinement of feature distributions in complex scenes.

7. any specific Reason for 67% accuracy for Known images and 89% for unknown images. Usually Known images will have higher accuracy.

Reply: Thank you for your review comments. This result is mainly due to the effect of the dynamic threshold adjustment strategy. Specifically, during the dynamic adjustment process, the study set the threshold as a balance point between the classification performance of known and unknown images to maximize the model's ability to generalize to unknown images. When the threshold is adjusted to 0.6, the test results reach the intertwining point, i.e., the model's classification performance on known and unknown images tends to be balanced, and the accuracy rate of known images, although slightly lower, is significantly improved to 89% for unknown images. The main purpose of this strategy is to optimize the model's ability to recognize unknown categories under open-set conditions, reflecting the research's design principle of prioritizing the classification of unknown images so as to better meet the needs of the open-domain adaptation task.
