# Supplementary material for: Application of open domain adaptive models in image annotation and classification
Source: PLoS One. 2025 May 14;20(5):e0322836. doi: 10.1371/journal.pone.0322836 (PMC12077773; doi:10.1371/journal.pone.0322836)
Supplement: Attachment — Submitted filename: Response_to_reviewers_comments_auresp_2.docx [file pone.0322836.s004.docx]

Review Comments to the Author

Please use the space provided to explain your answers to the questions above. You may also include additional comments for the author, including concerns about dual publication, research ethics, or publication ethics. (Please upload your review as an attachment if it exceeds 20,000 characters)

Reviewer #1: Please consider the following....

To make the model’s generalization more interpretable, uncertainty estimation methods like Monte Carlo Dropout or Bayesian Neural Networks can be used to show the model's confidence in predicting unknown classes. This helps in understanding how the model handles open-set situations.

Reply: Thank you very much for your comments. In this paper, (like Monte Carlo Dropout) to show the model's confidence in predicting unknown classes (Table 1). Please see details as follows:

Monte Carlo culling is also used to estimate the confidence of the different models to assess their reliability and generalization ability in cross-domain data migration. The confidence score reflects the confidence level of the model in dealing with unknown categories, which enhances the interpretability of the model. The test results are shown in Table 1.

**Table 1 Test results of indicators for different models**

| Categorized Object | Model | P/% | R/% | F1/% | Running Time/s | Annotation Consistency | Efficiency/% | Confidence score |
| --- | --- | --- | --- | --- | --- | --- | --- | --- |
| Known Areas | DANN | 78.6 | 81.2 | 79.9 | 4.2 | 0.86 | 89.72 | 0.79 |
|  | CADA | 83.5 | 85.7 | 84.6 | 5 | 0.81 | 87.34 | 0.82 |
|  | SEDA | 79.6 | 85.1 | 82.3 | 3.1 | 0.89 | 89.19 | 0.85 |
|  | ODA-IAC | 89.6 | 90.5 | 90 | 1.8 | 0.91 | 91.28 | 0.91 |
| Unknown Areas | DANN | 82.1 | 84.6 | 83.3 | 4.2 | 0.85 | 79.34 | 0.76 |
|  | CADA | 83.4 | 85.7 | 84.5 | 4.1 | 0.89 | 83.52 | 0.81 |
|  | SEDA | 79.6 | 87.9 | 83.7 | 2.5 | 0.91 | 84.41 | 0.87 |
|  | ODA-IAC | 89.3 | 90.7 | 90.2 | 1.2 | 0.92 | 89.56 | 0.93 |

In the unknown domain, i.e., data that are unlabeled or not present at all in the training data of the source domain, the checking accuracy and recall are 89.3% and 90.7%, respectively, with an F1 value of 90.2%, and the annotation consistency is further improved to 0.92, with the shortest running time of only 1.2 seconds.

Meanwhile, the confidence analysis further verifies the stability and reliability of the ODA-IAC model in dealing with unknown categories, and its confidence scores reach 0.91 and 0.93 in the known and unknown domains, respectively, which are significantly higher than those of the other models, indicating that the model is not only able to provide high-precision categorization results, but also make predictions with higher confidence, which is crucial for reducing the risk of misclassification. In contrast, the confidence scores of DANN and CADA are 0.76 and 0.81, respectively, which are significantly less adaptable in the unknown domain, while the confidence score of SEDA reaches 0.87 but is still lower than that of ODA-IAC, suggesting that its handling of cross-domain data migration still has some limitations.

Visualizing decision boundaries using techniques like SHAP or LIME can clarify how the model classifies images, particularly when adapting to domain shifts in open-set scenarios. The authors could also show how domain alignment affects the decision boundary or how features from the source domain are adapted for the target domain.

Reply: Thank you very much for your comments. SHAP and LIME have been used to clarify the images classification by the model. The decision boundary affected by domain alignment and the features from the source domain adapted to the target domain have been explained. Please see details as follows:

**Model image classification by SHAP and LIME:**
In order to gain a deeper understanding of how models classify in open-set scenarios, especially how they adapt to the transfer between source and target domains, the study introduces SHAP (SHapley Additive exPlanations) and LIME (Local Interpretable Model-agnostic Explanations) techniques to visualize the decision boundary for image classification, respectively. With SHAP and LIME, the study is able to accurately track how the model assigns different weights to different features when classifying images in the target domain, and is able to visualize the model's behavioral patterns when adapting to unknown categories. In particular, during the process of domain transfer, the model optimizes the classification performance by adjusting the decision boundary to ensure an appropriate response to the feature differences between the source and target domains.

**The decision boundary affected by domain alignment:**

As can be seen in Fig. 12, it is found that the classification decision boundary is looser when the methods of L2T and JAN are used, and the feature distributions of some target domain samples and source domain samples are not fully aligned, resulting in more misclassification cases of unknown categories. While the ADDA model has some improvement in domain alignment, its classification boundary still has a local offset, especially on the unseen category, and the feature mapping is more ambiguous. In contrast, the visualization results of the research-proposed ODA-IAC model on the VisDA dataset show that the domain alignment strategy significantly optimizes the classification boundaries, resulting in a tighter aggregation of features in similar categories and a more discrete distribution of features in different categories, which improves the model's adaptive ability on the target domain. **The features from the source domain adapted to the target domain:**

Furthermore, during the feature adaptation process between the source and target domains, the model aligns the data distributions of the source and target domains by introducing DA. Specifically, the features of the source domain are extracted and mapped to the feature space of the target domain through a feature generator and a channel attention mechanism. This process ensures that the features learned in the source domain can be effectively applied in the target domain by minimizing the feature differences between the source and target domains. To further optimize this adaptation process, the study employs SAS, which adapts the source domain features at a finer granularity by dividing the extensive source or target domains into multiple subdomains, allowing the features of the target domain to match the source domains more accurately and reducing the inter-domain distributional differences.

Reviewer #2: The authors responded for the quires. The review responses are satisfactory. The revised manuscript can be accepted for publication.

Reply: Thank you very much for your satisfaction with the author's revisions to this paper and your kind recommendation (accept).
